# Spectrophotometric Determination of Labetalol and Lercanidipine in Pure Form and in Pharmaceutical Preparations Using Ferric-1,10-Phenanthroline

**Published:** 2009-09

**Authors:** M. A. Abu El-Enin, D. R. El-Wasseef, D. T. El-Sherbiny, S. M. El-Ashry

**Affiliations:** *Department of Medicinal Chemistry, Faculty of Pharmacy, University of Mansoura, Mansoura, Egypt*

**Keywords:** Ferric-1,10-Phenanthroline, labetalol, lercanidipine, spectrophotometry

## Abstract

A simple and sensitive spectrophotometric method was developed for the determination of labetalol HCl (LBT) and lercanidipine HCl (LER) in pure form and in dosage forms. The method was based upon oxidation of the LBT and LER with Fe^+3^ and the estimation of the produced Fe^+2^ with 1,10-phenanthroline. The absorbance of the tris(1,10-phenanthroline) Fe^+2^ complex was measured at 510 nm. Reaction conditions were optimized to obtain colored complex of higher sensitivity and longer stability. The absorbance concentration plots were rectilinear over the concentration rang of 5–90 and 1–20 μg/mL with lower detection limits of 0.74 and 0.01 μg/mL and quantification limits of 2.26 and 0.02 μg/mL for LBT and LER, respectively. The developed method was successfully applied for the determination of LBT and LER in bulk drugs and dosage forms. The common excipients and additives did not interfere in their determinations. There was no significant difference between the results obtained by the proposed and the reference methods regarding Student t-test and the variance ratio F-test.

## INTRODUCTION

Labetalol HCl: 5-[1-hydroxy-2-(1-methyl-3-phenylpropylamino)ethyl] salicylamide hydrochloride. LBT is a non-cardiovascular β-blocker. It is used in the management of hypertension and to induce hypotension during surgery (1). LBT is the subject of a monograph in each of the British Pharmacopoeia, BP (2) and the United States Pharmacopoeia, USP (3). The BP recommends non aqueous titration for the raw material and spectrophotometric measurement at 302 nm for the tablets and injections. The USP (3), on the other hand, recommends HPLC method for the raw material and its formulations.

The therapeutic importance of LBT initiated several reports on its determination in formulations *viz:* spectrophotometry (4–7), spectrofluorimetry (8–13), HPLC (14–17), HPLC-MS (18–20), capillary electrophoresis (21, 22), and Voltammetry (23). LBT was also determined in pharmaceuticals using an ion selective electrode sensitive to LBT with a liquid membrane (24).

Lercanidipine HCl: (±)-2-[(3,3-Diphenylpropyl)methylamino]-1,1-dimethylethyl methyl-,4-dihydro-2,6-dimethyl-4-(*m*-nitrophenyl)-3,5-pyridine dicarboxylate hydrochloride.

LER is a dihydropyridine calcium-channel blocker with actions similar to those of nifedipine. It is used in the treatment of hypertension (1).

There are several reports on the determination of LER, *viz:* spectrophotometry (25–30), voltammetry (31, 32), capillary electrophoresis (33) and several HPLC methods (34–43).

The main goal of the study is to develop an accurate, simple and non expensive spectrophotometric method for the determination of LBT and LER in pure form and in pharmaceutical preparations.

## EXPERIMENTAL

### Materials

LBT pure sample was purchased from Sigma (St. Louis, Mo, USA) and used as received. LER was kindly provided by Recordati Industria Chimica e Farmaceutica S.p.A. via Mediana Cistema, Milan, Italy (Lot No. 03000630) and was used as received. Tablets containing LER (Lercan) and LBT (Trandate) were obtained from commercial sources. Glucose, lactose, dextrose, sucrose, starch, cetrimide, sodium dioctyl sulfosucinate (SDOSS), sodium dodecyl sulfate (SDS), 3-(N,N-dimethymyristyl)ammoniopropane sulfonate (MAPS) and polyoxyethylene 23 lauryl ether (Brij 35) were purchased from Sigma (St. Louis, Mo, USA).

### Reagents

Ferric-1,10-phenanthroline phenanthroline reagent (FPL) was prepared by mixing 0.193 g of 1,10-phenanthroline (Sigma-Aldrich, St. Louis, USA) with 2 mL of 1.0 M HCl and 0.16 g of ferric ammonium sulfate dodecahydrate (Sigma-Aldrich St. Louis, USA)and diluted with distilled water to 100 mL (58).

### Apparatus

A Shimadzu UV-Visible 1601 PC spectrophotometer with 1 cm quartz cells was used for recording spectra and absorbance measurements.

### Standard Solutions

Stock solutions containing 1.0 mg/mL LBT and LER were prepared separately in methanol. The stock solution of LBT was used as a working solution while the stock solution of LER was further diluted with methanol to 200.0 μg/mL. The standard solutions were found to be stable for at least one week when protected from light and kept in the refrigerator.

## METHODS

### Recommended procedures

Accurately measured aliquots containing LBT and LER covering the final concentration range cited in Table 1 were transferred into a set of 10 mL stoppered volumetric flasks. Three mL of FPL in the case of LBT and 2.0 mL in the case of LER were added, the flasks were stoppered and heated in a boiling water bath for 25 and 15 min in the case of LBT and LER, respectively. The reaction mixture was allowed to cool, and then the volume was adjusted to the mark with distilled water. The absorbance was measured at 510 nm against a reagent blank. The absorbance was plotted *vs*. final concentration of the drug (μg/mL) to get the calibration graph. Alternatively, the corresponding regression equations were derived.

### Applications for pharmaceutical formulations

Accurately weighed amounts of the powdered Trandate^®^ and Lercan^®^ tablets equivalent to 100.0 mg of LBT and 20.0 mg of LER, respectively, were transferred into separate small conical flasks, 50.0 mL methanol were added and the solutions were sonicated for 10 min; then filtered into 100 mL volumetric flasks. The conical flasks were washed with few milliliters of methanol; the washings were passed into the corresponding volumetric flasks which were then completed to the mark with methanol to give a working solution of 200.0 μg/mL and 1000.0 μg/mL for LER and LBT, respectively. Aliquots in the concentration range cited in Table 1 were transferred into 10.0 mL volumetric flasks. The general procedure was then applied as under construction of calibration graph, and the nominal contents of tablets were determined either from a previously plotted calibration graphs or using the corresponding regression equations.

## RESULTS AND DISCUSSION

Ferric salts play an important role in the spectrophotometric determination of many pharmaceutically important phenolic compounds. Acting as an oxidizing agent, Fe^+3^ is reduced by the drug to Fe^+2^ and its amount is proportional to the drug concentration. The amount of Fe^+2^ is determined using 1,10-phenanthroline.

**Figure 1:**
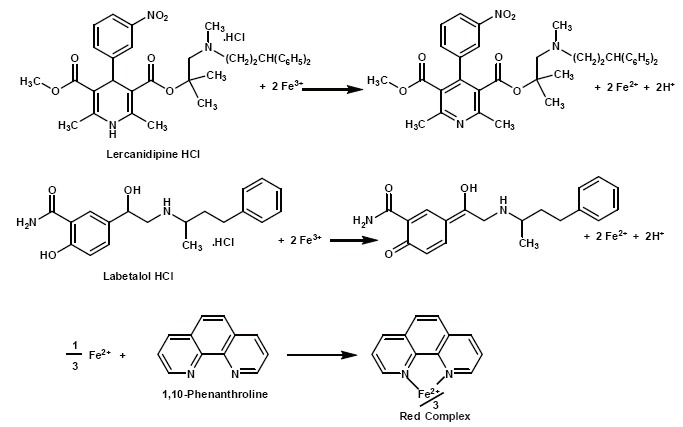
Proposal of the mechanism of reaction between labetalol and lercanidipine with ferric-1,10-phenanthroline.

In this study, Fe^+3^ oxidizes the phenolic moiety of LBT and the dihydropyridine ring of LER and the produced Fe^+2^ forms a red colored complex, tris(1,10-phenanthroline)Fe^+2^, with 1,10-phenathroline which exhibits an absorption band peaking at 510 nm. The proposed mechanism of action was shown in Fig. 1.

The optimum conditions for color development were established by varying one parameter at a time, keeping the others fixed and observing the effect produced on the absorbance of the colored species.

### Volume of FPL

**Figure 2:**
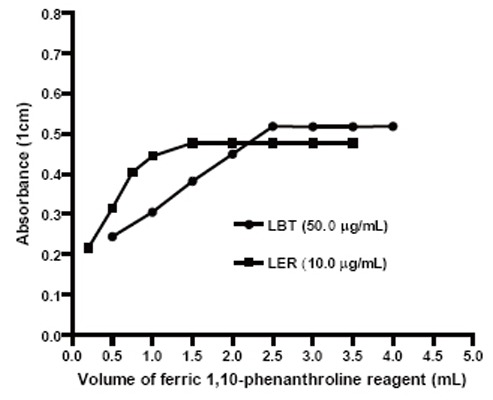
Effect of different volumes of 0.01 M ferric 1,10-phenanthroline reagent on the development of the reaction products.

The effect of the reagent was studied by measuring the absorbance of the solution containing a fixed concentration of LBT and LER and varied amount of the reagent separately. Constant and maximum color development of the complex was achieved with a reagent volume of 2.5 and 1.5 mL for LBT and LER, respectively (Fig. 2). Although a larger volume of the reagent had no effect on the complex formation, the absorbances increased slightly due to background of the colored reagent. However, 3.0 and 2.0 mL of FPL for LBT and LER, respectively, were used to ensure complete reaction.

### Heating temperature

**Figure 3:**
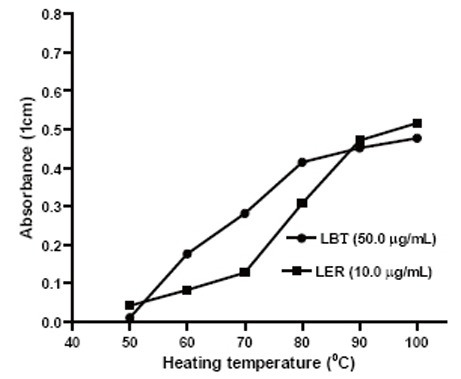
Effect of the heating temperature on the formation of the colored product.

**Figure 4:**
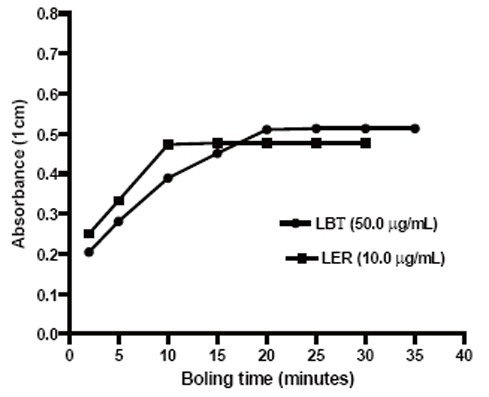
Effect of boiling time on the development of the reaction products.

The formation of colored complex was very slow at room temperature and required longer time for completion. Hence, efforts were made to accelerate by carrying out the reaction at higher temperatures. It was observed that the maximum absorbances were obtained after heating the reaction mixture at 100°C (15 and 25 min for LER and LBT, respectively) (Fig. 3 and Fig. 4). The absorbance of the complex remained constant at room temperature for more than 4.0 hours.

### Effect of Common Excepients and Different surfactants

The effect of common excipients and additives (e.g. glucose, starch, lactose and dextrose) was tested for their possible interferences in the assay of LBT and LER. In the same manner, the effect of different surfactants [cationic surfactant e.g. cetrimide, anionic surfactant e.g. sodium dioctyl sulfosucinate (SDOSS) and sodium dodecyl sulfate (SDS) and nonionic surfactants e.g. 3-(N,N-dimethymyristyl) ammoniopropane sulfonate (MAPS) and polyoxyethylene 23 lauryl ether (Brij 35)] on the absorbance of the formed complex was investigated by adding three different concentration of each surfactant to the reaction mixture. It was found that all of these tested substances do not interfere with the analysis by the proposed method.

### Validation of the proposed methods

The proposed method was valid with respect to linearity, limit of quantification (LOQ), limit of detection (LOD), accuracy, precision, and specificity:

**Linearity:** The absorbance-concentration plots for the studied drugs were linear over the concentration range cited in Table 1. Linear regression analysis data are given in Table 1

**Table 1:** Performance data for the spectrophotometric determination of LBT and LER using FBL reagent

Parameters	LBT	LER

Concentration range (μg/ml)	5.0–90.0	1.0–20.0
Apparent molar absorptivity (L/mol/cm)	4.44 × 10^3^	3.29 × 10^4^
${\text{A}}_{1\text{cm}}^{1\text{\%}}$	121.66	507.42
Correlation coefficient	0.9999	0.9999
Slope	0.07	0.03
Intercept	0.01	0.05
LOD (μg/ml)	0.74	0.01
LOQ (μg/ml)	2.26	0.02
S_y/x_	3.36 × 10^−3^	1.39 × 10^−3^
S_a_	2.03 × 10^−3^	8.48 × 10^−3^
S_b_	37.99 × 10^−3^	6.19 × 10^−3^
% RSD	0.81	0.75
% Er	0.26	0.24

LOD, limit of detection; LOQ, Limit of quantification S_y/x_, standard deviation of the residuals; S_a_, standard deviation of the intercept; S_b_, standard deviation of the slope; RSD%, relative standard deviation; Er%=RSD/√n.

**Limit of Quantification (LOQ) and Limit of Detection (LOD):** The limit of quantification (LOQ) was determined by establishing the lowest concentration that could be measured according to ICH Q2(R1) recommendations (45), below which the calibration graph is non linear (LOQ=10σ/S where S is the slope and σ is the standard deviation of the intercept of regression line of the calibration curve). The limit of detection (LOD) was determined by evaluating the lowest concentration of the analyte that can be readily detected (LOD=3.3σ/S). The results of LOD and LOQ of LER by the proposed method are abridged in Table 1.

**Accuracy and precision:** The results of the inter-day and intra-day accuracy and precision of the method are summarized in Table 2. The inter-day and intra-day precisions were examined by analysis of LBT in concentrations 10.0, 40.0 and 70.0 µg/mL and LER in concentrations 5.0, 10.0 and 15.0 µg/mL each three times a day for three consecutive days. The precision of the proposed method is fairly high, as indicated by the low values of SD and %RSD, respectively. Also the inter-day and intra-day accuracy was proved by the low values of %Er.

**Table 2:** Evaluation of the accuracy and precision data of the proposed spectrophotometric method for the determination of LBT and LER

Drug	Amount added (μg/ml)	Amount found (μg/ml)^a^	found ± SD %	RSD %	Er %

LBT	Intra-day				
	10.00	9.92	99.54 ± 0.46	0.46	0.27
	40.00	39.88	99.67 ± 0.84	0.84	0.49
	70.00	70.89	100.17 ± 0.55	0.55	0.32
	Inter-day				
	10.00	9.94	99.40 ± 0.88	0.89	0.51
	40.00	40.87	101.05 ± 0.98	0.97	0.56
	70.00	69.90	99.36 ± 0.55	0.55	0.32
LER	Intra-day				
	5.00	5.00	100.00 ± 0.72	0.72	0.42
	10.00	9.98	99.83 ± 0.91	0.91	0.53
	15.00	14.96	99.74 ± 0.68	0.68	0.39
	Inter-day				
	5.00	5.00	100.07 ± 0.76	0.76	0.44
	10.00	9.99	99.87 ± 0.38	0.38	0.22
	15.00	15.10	100.64 ± 0.30	0.30	0.17

a Each result is the average of three separate experiments.

**Robustness of the method:** The robustness of the proposed method was demonstrated by the constancy of the absorbance with the deliberated changes in the experimental parameters such as volume of FPL, 3.00 ± 0.20 and 2.00 ± 0.20 mL for LBT and LER, respectively, and heating time 25.00 ± 2.00 15.00 ± 2.00 min. for LBT and LER, respectively. These minor changes that may take place during the experimental operation didn't greatly affect the absorbance of the formed complex.

### Application to tablets analysis

The proposed method was applied to the determination of the studied drug in their commercial preparations. The selectivity of the method was investigated by observing any interference encountered from the common tablet excepients. These excepients did not interfere with the proposed method. The results of the proposed method were compared with those obtained using the comparison method (13, 29). Statistical analysis (44) of the results obtained using Student's t-test and variance ratio F-test revealed no significant difference between the performance of the two methods regarding the accuracy and precision, respectively, Table 3.

**Table 3:** Application of spectrophotometic method for the determination of LBT and LER in tablets

Preparation	% Found ± SD	
	Proposed method	Reference method (13, 29)

Trandate^®^ a tablets (100.0 mg LBT/tablet)	99.85 ± 0.56	99.92 ± 0.61
	*t*-value 0.25 (2.45)	
	*F*-value 1.19 (9.28)	
Trandate^®^ a tablets (200.0 mg LBT/tablet)	100.11 ± 0.93	100.36 ± 0.64
	*t*-value 0.53 (2.45)	
	*F*-value 2.11 (9.28)	
Lercan^®^ b tablet (10.0 mg LER/tablet)	100.18 ± 0.43	100.41 ± 0.76
	*t*-value 1.02 (2.45)	
	*F*-value 3.12 (9.28)	
Lercan^®^ b tablet (20.0 mg LER/tablet)	99.95 ± 0.68	100.408 ± 0.765
	*t*-value 1.24 (2.45)	
	*F*-value 1.25 (9.28)	

Four independent analyses. Values between brackets are the tabulated *t* values and *F*-values at (P=0.05).

a Product of Glaxo Smith-Kline. Lot No. 825317 and 945001;

b Product of Laboratories Recordati. Industria Chimica e Farmaceutica S.p.A. *via* Matteo Civitali, 1-20148 Milan-Italie. Lot No. M06E07 and MO8C18.

## CONCLUSION

The higher λ_max_ of the proposed visible spectrophotometric method over the reported UV and visible spectrophotometric method is decisive and advantageous since interference from the excipients should be far less at higher wavelengths. The proposed method was accurate and precise as indicated by good recoveries of the drugs and low RSD values. Although the proposed method is time consuming it was found to be more sensitive in comparison with the reported method. Also the proposed method can be applied for routine analysis and in quality control laboratories for quantitative determination of the cited drugs both in the pure and dosage forms.
